# High Behavioral Variability Mediated by Altered Neuronal Excitability in *auts2* Mutant Zebrafish

**DOI:** 10.1523/ENEURO.0493-20.2021

**Published:** 2021-10-08

**Authors:** Urvashi Jha, Igor Kondrychyn, Vladimir Korzh, Vatsala Thirumalai

**Affiliations:** 1 National Centre for Biological Sciences, Tata Institute of Fundamental Research, Bangalore 560065, India; 2 School of Chemical and Biotechnology, SASTRA Deemed University, Thanjavur 613401, India; 3 Institute of Molecular and Cell Biology, Proteos 138673, Singapore; 4 International Institute of Molecular and Cell Biology, Warsaw 02-109, Poland

**Keywords:** C-start, calcium imaging, escape behavior, intrinsic properties, Mauthner, transcription

## Abstract

Autism spectrum disorders (ASDs) are characterized by abnormal behavioral traits arising from neural circuit dysfunction. While a number of genes have been implicated in ASDs, in most cases, a clear understanding of how mutations in these genes lead to circuit dysfunction and behavioral abnormality is absent. The *autism susceptibility candidate 2* (*AUTS2*) gene is one such gene, associated with ASDs, intellectual disability and a range of other neurodevelopmental conditions. However, the role of AUTS2 in neural development and circuit function is not at all known. Here, we undertook functional analysis of Auts2a, the main homolog of AUTS2 in zebrafish, in the context of the escape behavior. Escape behavior in wild-type zebrafish is critical for survival and is therefore, reliable, rapid, and has well-defined kinematic properties. *auts2a* mutant zebrafish are viable, have normal gross morphology and can generate escape behavior with normal kinematics. However, the behavior is unreliable and delayed, with high trial-to-trial variability in the latency. Using calcium imaging we probed the activity of Mauthner neurons during otic vesicle (OV) stimulation and observed lower probability of activation and reduced calcium transients in the mutants. With direct activation of Mauthner by antidromic stimulation, the threshold for activation in mutants was higher than that in wild-type, even when inhibition was blocked. Taken together, these results point to reduced excitability of Mauthner neurons in *auts2a* mutant larvae leading to unreliable escape responses. Our results show a novel role for Auts2a in regulating neural excitability and reliability of behavior.

## Significance Statement

Autism susceptibility candidate 2 (AUTS2) is one among recently identified autism susceptibility candidate genes, whose function in neuronal circuits is unclear. Using zebrafish as a model organism, we probe the function of Auts2a (homolog of mammalian AUTS2) at the cellular, network and behavioral levels. The escape behavior of *auts2a* mutant zebrafish is highly variable with normal short latency escapes, long latency escapes and total failures across trials in the same fish. This occurs because neuronal excitability is inappropriately set in the Mauthner neurons of mutants leading to large trial-to-trial variability in responses. The behavioral variability is fully explained by variability in firing action potentials in the Mauthner neuron, providing an integrative understanding of how behavioral variability arises from mutations at the genetic level.

## Introduction

Every neuron needs to carefully tune its excitability to be able to perform computation within the circuit in which it is embedded. When firing properties are improperly specified, neuronal function is compromised leading to abnormal behaviors. Transcriptional regulation plays important roles in specifying neuronal excitability properties and therefore, are also a chief class of genes implicated in many neurologic diseases including autism spectrum disorders (ASDs; De Rubeis et al., 2014; Bourgeron, 2015; Sztainberg and Zoghbi, 2016). The *autism susceptibility candidate 2* (*AUTS2*; also known as activator of transcription and developmental regulator) gene is a known regulator of transcription in the nervous system (Gao et al., 2014; Russo et al., 2018; Wang et al., 2018) and is associated with several neurodevelopmental disorders including ASD (Sultana et al., 2002; Kalscheuer et al., 2007; Beunders et al., 2013). AUTS2 is expressed in neurons of the central nervous system and is present both in the nucleus and in the cytoplasm (Bedogni et al., 2010; Gao et al., 2014; Hori et al., 2014; Hori and Hoshino, 2017). In the nucleus, AUTS2 binds to members of the polycomb repressor complex 1 (PRC1) but activates transcription of several genes important for neural development and function (Gao et al., 2014; Oksenberg et al., 2014; Wang et al., 2018). In the cytoplasm, AUTS2 regulates the actin cytoskeleton to control neuronal migration and neurite outgrowth (Hori et al., 2014; Hori and Hoshino, 2017). *Auts2* knock-out (KO) mice exhibited several deficits such as reduced righting reflexes and ultrasonic vocalizations (Gao et al., 2014). Nevertheless, how AUTS2 controls nervous system development, function and behavioral output are not understood at all.

Previous studies have identified and characterized four paralogs of *auts2* in zebrafish: *auts2a*, *auts2b*, *fibrosin-like 1* (*fbrsl1*), and *fibrosin* (*fbrs*; Kondrychyn et al., 2017). Both *auts2a* and *auts2b* genes are expressed in the developing and juvenile zebrafish brain. Analysis of gene structure and protein sequence revealed that among the two genes, *auts2a* is the closest orthologue to mammalian *Auts2* (61.58% identity in protein sequence), with even higher homology in the C terminus, hinting at conserved binding partners and function. In larval zebrafish, *auts2a* is widely expressed in the brain with distinctly high expression in rhombomere 4 (Kondrychyn et al., 2017), which houses neurons of the escape network (Metcalfe et al., 1986).

Teleost escape behavior consists of a sharp C-shaped tail bend away from the inducing stimulus with only a few milliseconds latency (Kimmel et al., 1974; Eaton et al., 1977). The behavior is triggered by action potential firing in one of two bilaterally located giant Mauthner neurons (M-cells; Eaton and Farley, 1975; Eaton et al., 1977; Korn and Faber, 2005; Kohashi and Oda, 2008; Sillar, 2009). Two pairs of homologous neurons, MiD2cm and MiD3cm, also take part in escape behaviors but fire at much longer latencies (Eaton et al., 1984; Kohashi and Oda, 2008).

Action potential firing in M-cells is required for the fast C-start response (Zottoli, 1977; Eaton et al., 1981). In response to supra-threshold depolarization via strong synaptic inputs or by direct current injection, M-cells in larval and adult zebrafish, as well as adult goldfish, generate a single action potential with a very short latency (Eaton et al., 2001; Nakayama and Oda, 2004; Watanabe et al., 2014). This action potential is conducted quickly via its giant axons to spinal circuitry, including direct synapses onto contralateral motor neurons, resulting in rapid muscle contraction and a sharp bending of the body (Fetcho, 1991). M-cell excitability is thus critical for quick escape from threatening stimuli. Although not all of the conductances driving M-cell response have been delineated, it is clear that during development, M-cell intrinsic properties are progressively tuned to result in its mature firing behavior (Brewster and Ali, 2013; Watanabe et al., 2014, 2017). Since *auts2a* is expressed in rhombomere 4 at stages when M-cell properties are being defined, we sought to determine how Auts2a impacts excitability of M-cells and therefore the escape behavior itself.

Using customized transcription activator-like effector nucleases (TALENs), we generated mutations in the *auts2a* locus and isolated an allele, which had a premature stop codon in the coding sequence. Using a combination of high-speed videography and *in vivo* calcium imaging, we show that escape behaviors become highly unreliable and slow in *auts2a* mutants and that this unreliability can be explained by the reduced excitability of M-cells. These results indicate a role for Auts2a in regulating neuronal excitability, an action by which Auts2a impacts behaviors significantly.

## Materials and Methods

### Fish care and use

Zebrafish (*Danio rerio*) of AB strain and Indian wild type were housed in aquarium tanks at 28.5°C with a 14/10 h light/dark cycle. Fish were maintained according to established protocols as previously described (Westerfield, 2000) in agreement with the Institutional Animal Ethics Committee and the Institutional Biosafety Committee. For fin amputation, fish were briefly anaesthetized in 0.01% Tricaine (MS-222, Sigma-Aldrich), the caudal fin was cut and fish were immediately returned to fresh water. Experiments were performed on 6–8 d postfertilization (dpf) larval zebrafish at room temperature. Larvae have not undergone sex specification at these stages. Larvae were maintained in 14/10 h light/dark cycle at 28°C in E3 medium (5 mm NaCl, 0.17 mm KCl, 0.33 mm CaCl_2_ and 0.33 mm MgSO_4_, pH 7.8). Larvae were treated with 0.003% 1-phenyl-2-thiourea in 10% Hanks’ saline at 24 h postfertilization (hpf) to remove pigments, for calcium imaging experiments.

### TALEN design, construction, and synthesis

**Figure 1.**
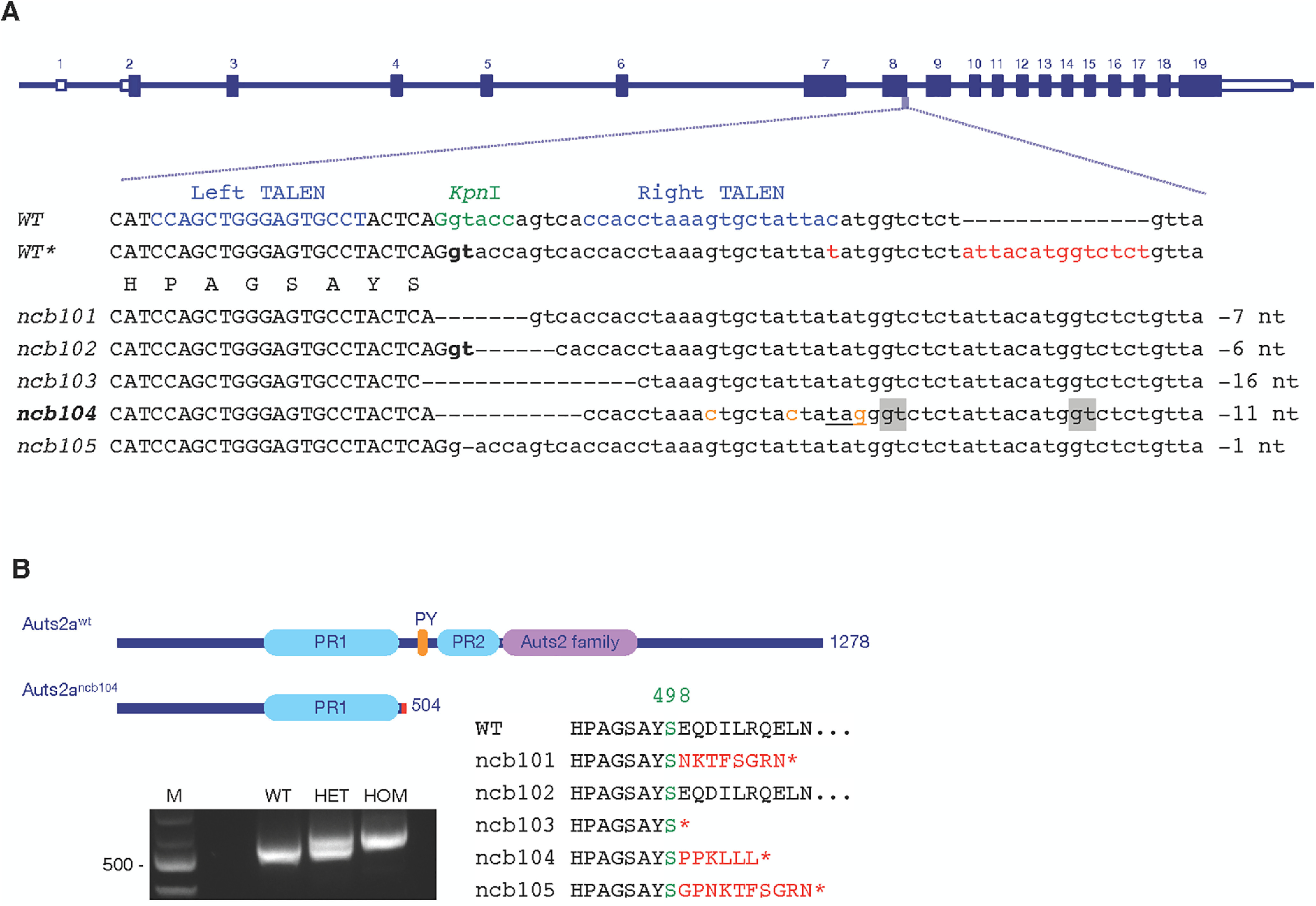
TALEN-induced mutation in the *auts2a* gene. ***A***, Zebrafish *auts2a* gene locus, TALEN target sites and isolated alleles. The TALENs target a pair of binding sites (in blue) flanking a spacer with a restriction enzyme site (in green). Exonic and intronic sequences are shown in upper and lower cases, respectively. In contrast to genomic sequence annotated in the Ensembl (WT), our “in-house” zebrafish AB strain (WT*) has a polymorphism in intronic sequence adjacent to exon 8 (in red). Alleles *ncb101*, *ncb103*, *ncb104*, and *ncb105* have the nucleotide deletions that disrupt the donor splice site (in bold) leading to a frameshift after S498 and premature stop codons. Deletion in allele *ncb102* does not affect correct splicing and the Auts2a protein sequence. Three single point mutations were introduced in the intron of *auts2a^ncb104^* allele (in orange) leading to a stop codon creation (underlined). The alternative donor splice sites used to splice mutant *auts2a^ncb104^* pre-mRNA are highlighted in gray. ***B***, top, Auts2a and Auts2a^ncb104^ proteins. The *ncb104* mutation causes the loss of the C-terminal portion of Auts2a, comprising PY motif, PR region PR2 and the Auts2 family domain. ***B***, bottom left, RT-PCR analysis of *auts2a* mRNA, isolated from wild-type (WT), *ncb104* heterozygote (HET), and homozygote (HOM) embryos. M, 100-bp DNA ladder (NEB). ***B***, bottom right, Partial protein sequences of mutant alleles. See also Extended Data Figure 1-1.

TALENs specific to *auts2a* were manually designed using criteria as described previously (Bedell et al., 2012) and assembled according to the established protocol (Sanjana et al., 2012). The plasmid kit used for building TALENs was a gift from Feng Zhang (Addgene kit #1000000019). Once assembled into a destination vector, TALENs were re-cloned into pTNT vector (Promega) containing synthetic poly A tail and T7 terminator sequence. The *auts2a* TALEN recognition sequences are as follows: left TALEN, CCAGCTGGGAGTGCCT and right TALEN, GTAATAGCACTTTAGGTGG. Between the two binding sites is a 16-nt spacer with a KpnI site (ACTCAGgtaccagtca, KpnI site is underlined, intronic sequence is in lower case), facilitating identification of mutations by PCR and KpnI restriction digestion. The spacer region overlaps with the donor splice site at exon 8 (Fig. 1*A*). Capped mRNA was synthesized by *in vitro* transcription using mMESSAGE mMACHINE T7 kit (Invitrogen) and purified with RNeasy Mini kit (QIAGEN). Zebrafish embryos at the one-cell stage were microinjected with 200-pg RNA (100 pg each of left and right TALEN mRNA). At such a dose, over 70% embryos survived and showed TALEN-induced somatic *auts2a* gene modifications.

10.1523/ENEURO.0493-20.2021.f1-1 Extended Data Figure 1-1 Auts2a^ncb104^ allele in different auts2a isoforms. ***A,*** Overview of transcripts generated from the *auts2a* gene (modified from Kondrychyn et al., 2017). Noncoding and coding exons are depicted as open and filled bars, respectively. Alternative transcription start sites are used to generate auts2a isoforms. ***B***, Schematic structure of Auts2a^wt^ and Auts2a^ncb104^ proteins translated from the different auts2a isoforms. Positions of coding exons are marked for the reference (relative exon size is not in scale). Missense amino acids preceding premature stop codon are shown in red. Exons 1B and 1D code the alternative N-terminal amino acids. PR1 spans exons 7 and 8, PR2 spans exons 9–13, and the Auts2 family domain spans exons 14–19. Download Figure 1-1, TIF file.

### DNA isolation and genotyping

Genomic DNA was isolated from either embryos or fin clips using HotSHOT method (Meeker et al., 2007). One microliter solution was then used in a 25-μl PCR containing the following reagents at these concentrations: 200 nm each gene-specific primers (forward 5′-TCAGCGAACCCTACAGCTTCACACA-3′ and reverse 5′-TGGGGTACGCACCATGGGCGGTGCA-3′), 0.2 mm dNTPs, 1× PCR buffer and 0.625 units One*Taq* HotStart DNA polymerase (New England BioLabs). Reaction was amplified using the following conditions: 94°C for 1 min; 40 cycles of 94°C for 20 s, 68°C for 1 min; followed by 68°C for 1 min. PCR products were purified using PCR purification kit (QIAGEN) and digested with restriction enzyme KpnI-HF (New England BioLabs) at 37°C for 60 min. The resulting reactions were loaded onto a 1.8% agarose gel and electrophoresed in 1× Tris-acetate-EDTA (TAE) buffer. Mutations were assessed by loss of restriction enzyme digestion. To verify mutations, the gel purified uncut PCR products were cloned into pCRII-TOPO vector (TOPO TA Cloning kit, Invitrogen) and sequenced.

### RT-PCR and RNA isolation

Total RNA was isolated from wild-type and *auts2a^ncb104^* heterozygote and homozygote embryos using an RNeasy Mini kit (QIAGEN), and first-strand cDNA was synthesized from 1 μg of total RNA by oligo(dT) priming using SMARTScribe Reverse Transcriptase (Clontech) according to the manufacturer’s protocol. Amplification of cDNA was performed using Herculase II Fusion DNA polymerase (Agilent). Identity of amplified PCR products was verified by direct sequencing.

### Whole-mount immunohistochemistry

Embryos were fixed in 4% paraformaldehyde at 4°C overnight, washed three times for 15 min in PBST (1 × PBS, 0.1% Tween 20) and permeabilized in 0.1% Triton X-100 in 0.1% sodium citrate for 30 min at 4°C. Then embryos were incubated for 2 h in 5% Blocking reagent (Roche) in MAB (150 mm maleic acid, pH 7.5, 100 mm NaCl, 0.1% Tween 20) at room temperature. Embryos were incubated with 3A10 antibodies (DSHB, 1:200) overnight at 4°C, washed four times for 30 min in MAB and incubated with HRP-conjugated goat anti-mouse F(ab)_2_ fragments (Molecular Probe, 1:500) for 6 h at room temperature or overnight at 4°C. Embryos were extensively washed in PBST, stained with 3,3’-diaminobenzidine (DAB) and washed several times in PBST. Embryos were kept in 50% glycerol in PBS at 4°C until further imaging.

### Head restrained preparation

For behavior and calcium imaging experiments, larvae were embedded in 2% low gelling agarose (Sigma-Aldrich). E3 medium was added after the agarose congealed. Agarose around the tail and the OV were removed for observing tail movements and application of water pulse for behavioral experiments.

### High-speed recording of escape response

Escape responses were evoked by applying a water pulse to the OV or to the tail at the level of cloaca, with a glass capillary (tip diameter 0.05–0.06 mm) mounted on a micromanipulator (Narishige). The water pulses were generated by a pressure pulse of 10-ms duration and pressure of 30 psi from a microinjection dispense system (Picospritzer III, Parker Hannifin). Videos were acquired at 1000 fps with a high-speed camera (Phantom Miro eX4, Vision Research) mounted on a stereo microscope (SZX16, Olympus) at 512 × 512-pixel resolution and 500-μs exposure time. Methylene blue (1%) was used to visualize the water jet for tracking its contact with the larva. Six trials (three on each side) were performed on each larva.

### Retrograde labeling of Mauthner neurons

Mauthner neurons were retrogradely labeled with fluorescent calcium indicator Oregon Green Bapta-1 dextran, 10,000 MW (Invitrogen) or Calcium Green dextran, 10,000 MW (Invitrogen) for calcium imaging experiments. For Mauthner and its homologs imaging, neurons were retrogradely labeled with tetramethylrhodamine dextran, 1000 MW (Invitrogen). Larvae were first anaesthetized in 0.01% MS-222 (Sigma-Aldrich) in E3 medium. 25% OGB-1/CGD/TMR-dextran in 10% HBSS (137 mm NaCl, 5.4 mm KCl, 0.25 mm Na_2_HPO_4_, 0.44 mm KH_2_PO_4_, 1.3 mm CaCl_2_, 1.0 mm MgSO_4_, and 4.2 mm NaHCO_3_) was pressure injected with a glass microcapillary into the spinal cord (at the level of cloaca) using a Picospritzer. After injection, larvae were allowed to recover in HBSS for >12 h.

### Electrical stimulation

Electric shock stimuli (40 μA, 1 ms) were delivered using a bipolar electrode (FHC) placed at the OV. The pulse was generated using ISO-Flex stimulus isolator (A.M.P.I.), triggered by pClamp (Molecular Devices). For antidromic stimulation of the M-axon, larvae were anaesthetized in 0.01% MS-222 (Sigma-Aldrich) and were pinned down through notochord using fine tungsten wire (California Fine Wire). The MS222 was then replaced by external solution (composition: 134 mm NaCl, 2.9 mm KCl, 1.2 mm MgCl_2_, 10 mm HEPES, 10 mm glucose, 2.1 mm CaCl_2_, and 0.01 mm D-tubocurarine; pH 7.8; 290 mOsm) and skin along the tail was carefully removed using forceps (Fine Science Tools). Muscles in a hemi-segment (between the 10th and 13th myotomes) were carefully removed to expose the spinal cord. The bipolar electrode was placed on top of the exposed spinal segment and brief electrical stimuli of increasing strengths (10 μA onwards, 1 ms in duration) were delivered.

### Calcium imaging

Calcium activity on electrical stimulation of OV/M-axon, in retrogradely labeled Mauthner neurons, was imaged at 35–51 frames per second using an EMCCD camera (Evolve, Photometrics) mounted on a compound microscope (BX61W1, Olympus) with a water-immersion objective (LUMPlanFL 60×) and Image-Pro Plus (Media Cybernetics) acquisition software.

### Drugs

A total of 50 μm strychnine (Sigma-Aldrich) and 100 μm gabazine (Sigma-Aldrich) was dissolved in external solution. Measurements were taken after 2 min of drug application.

### Analysis

Data were analyzed using MATLAB (The MathWorks) and Fiji (NIH).

### Behavioral analysis

**Figure 2.**
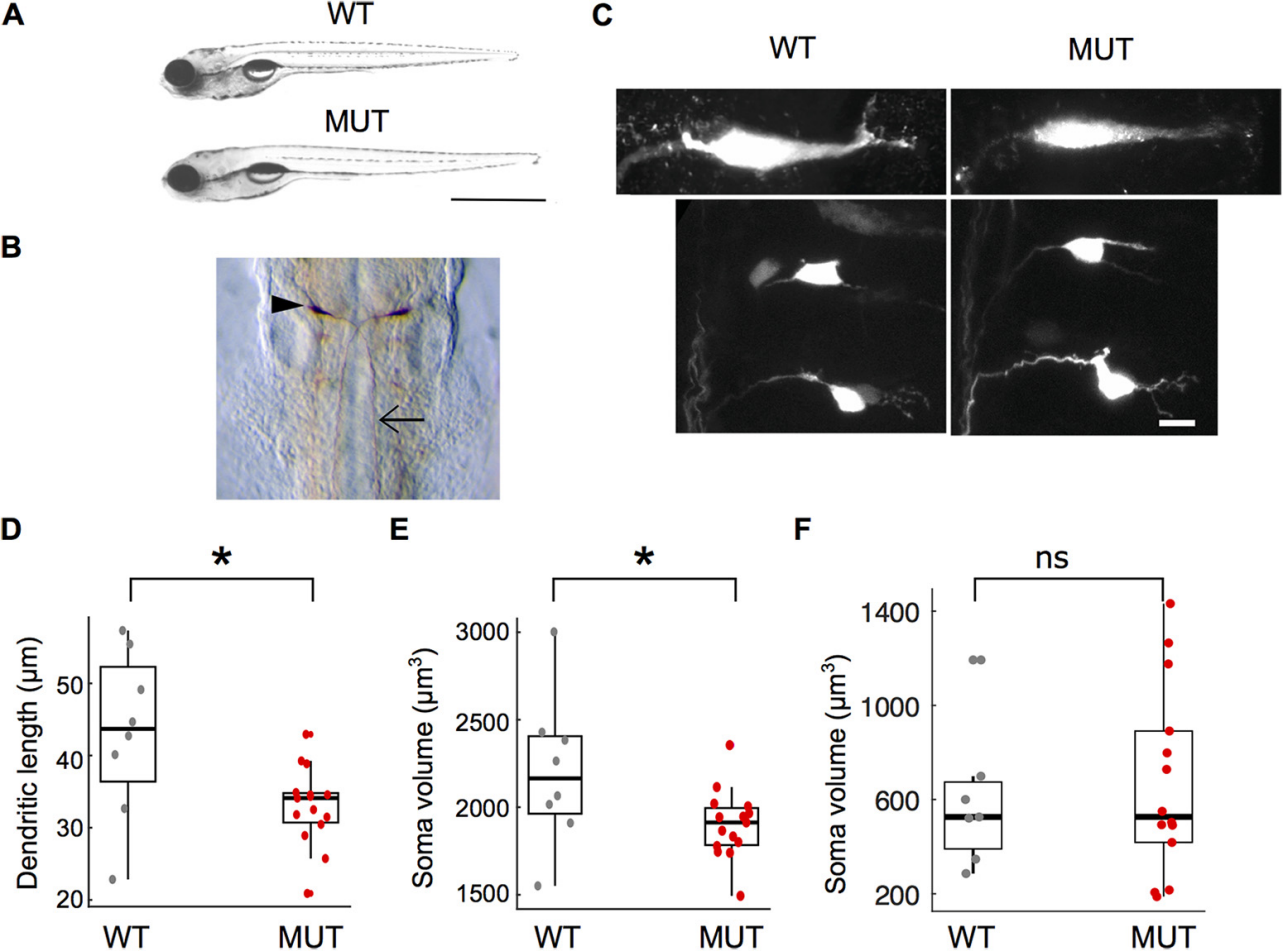
Morphologic characterization of *auts2a* mutants. ***A***, Bright field images of wild-type (WT) and mutant (MUT) larvae. Scale bar: 2 mm. ***B***, Whole-mount immunostaining with 3A10 antibody at 30 hpf in *auts2a* mutants. Arrowhead points to the cell body of the Mauthner neuron and arrow points to the axon. ***C***, Maximum intensity Z-projection of Mauther neuron (top) and homologs (bottom) of wild-type (left) and mutant (right) larvae. Scale bar: 10 μm. ***D***, Comparison of Mauthner lateral dendrite lengths in wild-type and *auts2a* mutant larvae. ***E***, Comparison of Mauthner soma volume in WT and mutant larvae. *n*_WT_ = 8 cells from 7 larvae; *n*_mut_ = 15 cells from 12 larvae; *p < 0.05; ns: not significant; Mann–Whitney *U* test. ***F***, Comparison of soma volume of the M-cell homologs. *n*_WT_ = 7 cells from 5 larvae; *n*_mut_ = 14 cells from 12 larvae. ns, not significant; Mann–Whitney *U* test.

Latency was defined as the time taken from when the water jet made contact with the larva to the first visible tail contraction. Tail bend angle was calculated by measuring the angle formed by joining a straight line passing through the tail at maximum bend and a straight line passing through the head and the tail in a prestimulus frame (Fig. 2*C*). Escape responses were defined as contralateral tail bends with latency ≤100 ms (Kimmel et al., 1974; Kohashi and Oda, 2008). Tail bend responses with latency >100 ms or no observable tail bend up to 1 s after stimulus delivery were classified as failures (“no response”).

### Calcium activity analysis

Relative changes in fluorescence from resting (ΔF/F) were calculated postbackground and movement correction. Activity in Mauthner neurons was considered to have occurred only for ΔF/F traces with minimum peak ΔF/F of 0.1 for OGB-1 dextran and 0.05 for CGD and full width at half maximum of at least 1 s. Threshold for antidromic stimulation of Mauthner was defined as the minimum stimulus intensity at which the Mauthner neuron showed calcium activity for at least three of five trials at the given stimulus intensity.

Dendritic length and soma volume were measured using simple neurite tracer and volume viewer plugins in Fiji (Schindelin et al., 2012). Dendritic length of the homologs could not be measured because of inadequate labeling given their small size.

### Statistics

Data were tested for normality using one sample Kolmogorov–Smirnov test (*p* < 0.05) and equality of variance with *F* test (*p* < 0.05). Two sample *t* test or Mann–Whitney *U* test were performed for comparisons between two groups and Kruskal–Wallis test was used for comparing three groups. χ^2^ test was performed for comparison of proportions. Table 1 presents a statistical summary of the data shown in this manuscript.

**Table 1:** Statistics

Figure number	Data structure	Type of test	Statistics
Figure 2*D*	Non-normal	Mann–Whitney *U* test	*p* = 0.0219 for differences in dendritic length between wild type and mutants
Figure 2*E*	Non-normal	Mann–Whitney *U* test	*p* = 0.0420 for differences in soma volume between wild type and mutants
Figure 2*F*	Normal	*t* test	*p* = 0.679 for differences in soma volume of homologs between wild type and mutants
Figure 3*C*	-	χ^2^ test	*p* = 6.5630e-09 and *p* = 6.2760e-15 for differences in contralateral tail bend between wild type and mutants and heterozygotes and mutants, respectively
Figure 3*C*	-	χ^2^ test	*p* =5.0549e-08 and *p* = 2.2116e-12 for differences in no tail bend responses between wild type and mutants and heterozygotes and mutants, respectively
Figure 3*C*	-	χ^2^ test	*p* = 2.2687e-06 and *p* = 0.0026 for differences in ipsilateral tail bend responses in wild type and mutant and heterozygotes and mutants, respectively
Figure 3*E*	Non-normal	Kruskal–Wallis; Mann–Whitney for between group comparisons	*p* = 1.0831e-06; *p* = 2.5258e-05 for differences in latencies between wild type and mutants, *p* = 8.7877e-06 for differences between heterozygotes and mutants.
Figure 3*F*	Non-normal	Kruskal–Wallis; Mann–Whitney for between group comparisons	*p* = 0.0004; *p* = 0.0010 for differences in latencies between wild type and mutants, *p* = 0.0010 for differences between heterozygotes and mutants
Figure 3*G*	Non-normal	Kruskal–Wallis; Mann–Whitney for between group comparisons	*p* = 0.0004; *p* = 6.3799e-04 for differences in CV of latencies between wild type and mutants, *p* = 5.7969e-04 for differences in CV between heterozygote and mutants
Figure 3*H*	Non-normal	Kruskal–Wallis; Mann–Whitney for between group comparisons	*p* = 0.0413; *p* = 0.9107 for differences in tail bend angle between wild type and mutants, *p* = 0.01 for differences between heterozygotes and mutants
Figure 4*C*	-	χ^2^ test	*p* = 0.0111 for differences in contralateral tail bend responses between wild type and mutants
Figure 4*C*	-	χ^2^ test	*p* = 1.4745e-06 for differences in no tail bend responses between wild type and mutants
Figure 4*C*	-	χ^2^ test	*p* = 0.3513 for differences in ipsilateral tail bend responses between wild type and mutants
Figure 4*D*	Normal	*t* test	*p* = 0.0044 for differences in Cumulative Density Function of latencies between wild type and mutants
Figure 4*E*	Normal	*t* test	*p* = 0.0676 for differences in CV of latencies between wild type and mutants
Figure 4*F*	Normal	*t* test	*p* = 0.0625 for differences in tail bend angle between wild type and mutants
Figure 5*D*	Non-normal	Mann–Whitney *U* test	*p* = 0.0079 for differences in probability of calcium activity in M-cell between wild type and mutants
Figure 5*E*	Non-normal	Mann–Whitney *U* test	*p* = 1.4939e-13 for differences in peak ΔF/F between wild type and mutants
Figure 5*F*	Non-normal	Mann–Whitney *U* test	*p* = 0.119 for differences in CV in ΔF/F between wild type and mutants
Figure 6*D*	Non-normal	Mann–Whitney *U* test	*p* = 0.0196 for differences in threshold between wild type and mutants
Figure 6*G*	Non-normal	Mann–Whitney *U* test	*p* = 2.8355e-04 for differences in peak ΔF/F between wild type and mutants
Figure 6*H*	Non-normal	Wilcoxon signed-rank test	*p* = 1 for before and after comparison for WT, *p* = 0.5 for before and after comparison for mutants
Figure 6*I*	Normal	*t* test	*p* = 0.1596 for before and after comparison for WT, *p* = 0.6467 for before and after comparison for mutants

## Results

### Generation of the *auts2a* KO zebrafish line

A pair of TALENs were designed to target the donor splice site at exon 8 of the *auts2a* gene (Fig. 1*A*). The TALEN-targeted sequences surround a restriction enzyme site for easy screening through introduction of a restriction fragment length polymorphism. We identified 5 different mutant alleles (Fig. 1*A*), three of which led to a frameshift after S498 and premature stop codons after several missense amino acids (Fig. 1*B*) and one of them, *auts2a^ncb104^,* was selected to establish an *auts2a* KO zebrafish line. This line harbors a 11-nt deletion, which disrupts the donor splice site affecting correct splicing between exon 8 and exon 9. RT-PCR analysis of RNA isolated from homozygotes revealed that *auts2a^ncb104^* pre-mRNA uses two alternative cryptic donor splice sites found in the intron to splice exon 8 to exon 9 (Fig. 1*A*,*B*). As a result, the intronic sequence is partially retained in *auts2a^ncb104^* mRNA leading to a premature stop codon at amino acid 504 after 6 missense amino acids (Fig. 1*B*).

The zebrafish Auts2a protein has several domains, previously predicted in human AUTS2 (Sultana et al., 2002): two proline-rich (PR) regions, PR1 at amino acids 273–492 and PR2 at amino acids 558–656, PY (PPPY) motif at amino acids 524–528, and the Auts2 family domain at amino acids 660–882 (Fig. 1*B*). In Auts2a^ncb104^, only the PR1 region is retained (Fig. 1*B*). The zebrafish *auts2a* gene locus shows tremendous transcriptional complexity with multiple isoforms generated via alternative splicing and alternative promoter usage (Kondrychyn et al., 2017). Exon 8 is a common exon in all isoforms (Extended Data Fig. 1-1*A*) and in *auts2a^ncb104^* mutant all isoforms will be similar affected: a loss of the C-terminal portion of Auts2a, comprising PY motif, PR2 region and the Auts2 family domain (Extended Data Fig. 1-1*B*).

### *auts2a* mutants display high variability in escape responses

*auts2a* KO zebrafish showed normal development and gross morphology (Fig. 2*A*). *auts2a^ncb104^* homozygote fish did not show significant difference in size [mean ± SD (mm): wild type, 5.0 ± 0.1 (24 larvae); mutants, 4.9 ± 0.2 (24 larvae); *p* = 0.37; Mann–Whitney test]. Moreover, *auts2a^ncb104^* homozygote fish survive to become fertile adults. As *auts2a* is a neurodevelopmental gene (Oksenberg and Ahituv, 2013) and is expressed at very high levels in rhombomere 4 (Kondrychyn et al., 2017), which houses the escape network, we first investigated whether M-cells, the command-like neurons driving escape behavior, are present in *auts2a* mutants. Wild-type larvae possess a single pair of M-cells that send commissural axons down the spinal cord (Fetcho, 1991). In *auts2a* mutants both M-cells are present and send commissural axons (Fig. 2*B*). However, M-cells in *auts2a* mutants were smaller: both soma volume and dendritic length were significantly reduced (Fig. 2*C*,*D*). No significant difference in soma volume was observed for the M-cell homologs (Fig. 2*C*,*E*). This suggests possible effects on M-cell function leading to deficits in escape behavior.

**Figure 3.**
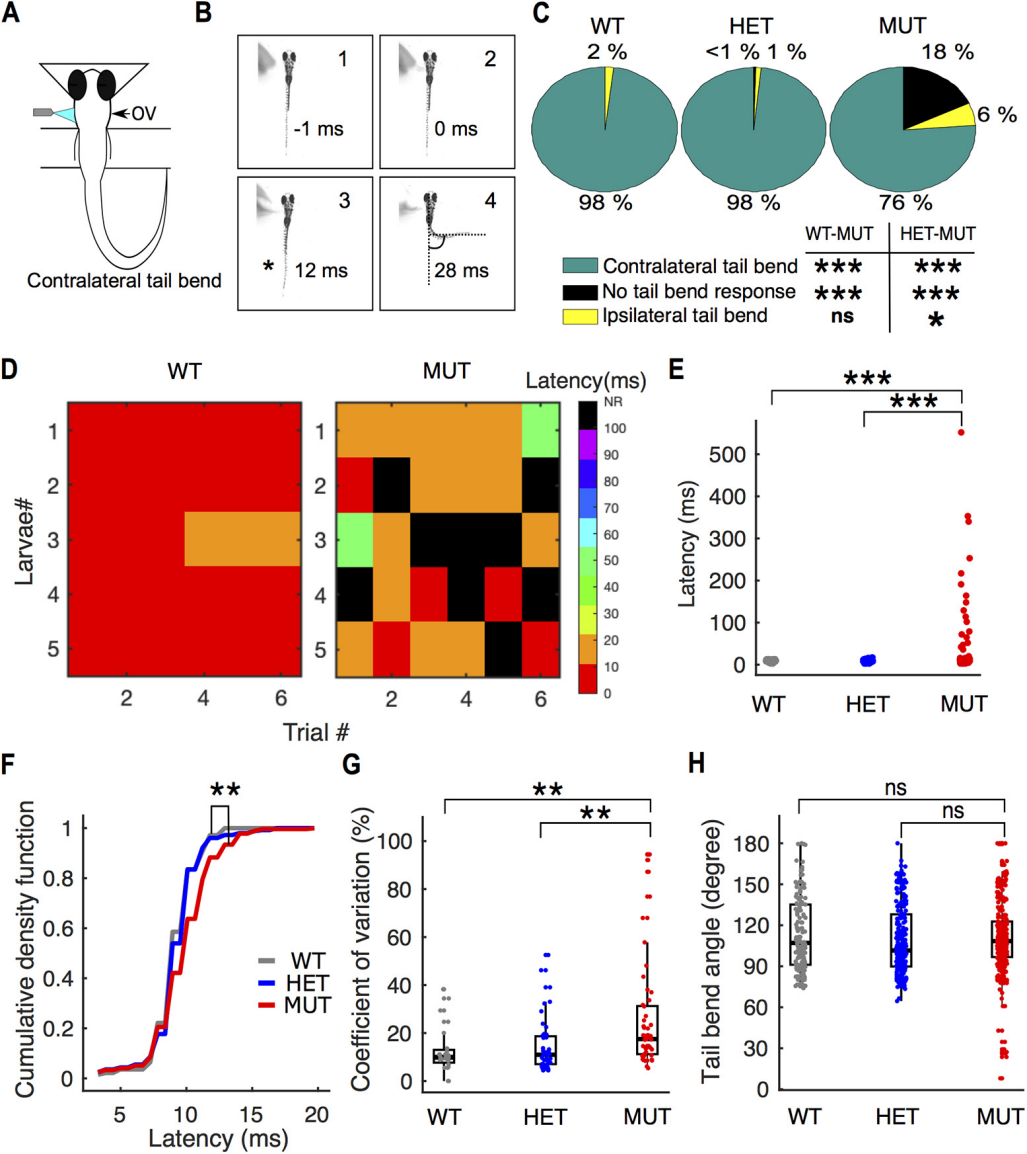
Onset of escape response is delayed and highly variable in *auts2a* mutants. ***A***, Schematic of experimental set up. Escape response was evoked in zebrafish larvae (6–8 dpf) by directing a strong water jet at the OV. Escape response is characterized by a large angle tail deflection, contralateral to the direction of water jet. ***B***, Time lapse of escape response. (1) Prestimulus frame. (2) Water jet makes first contact with OV. (3) First visible tail contraction (marked with asterisk). (4) Representative frame showing references used for maximum tail bend angle calculation. ***C***, Pie chart showing percentage of contralateral, ipsilateral, and no tail bend responses observed across wild type (*n* = 143 trials), heterozygotes (*n* = 258 trials), and *auts2a* mutants (*n* = 371 trials); χ^2^ test. ***D***, Escape latencies across successive trials from five wild-type and mutant larvae. Color bar represents escape latencies. NR: no response. ***E***, Comparison of escape response latencies in wild type (WT), heterozygotes (HET), and *auts2a* mutants (MUT). *n*_WT_ = 140 trials from 24 larvae, *n*_HET_ = 254 trials from 43 larvae and *n*_MUT_ = 292 trials from 57 larvae. ***F***, Cumulative density function plot for short-latency escapes (latencies ≤20 ms) in wild type (*n* = 140 trials), heterozygotes (*n* = 254 trials), and *auts2a* mutants (*n* = 273 trials). ***G***, CV of latencies across successive trials in individual larvae for wild type (*n* = 24), heterozygotes (*n* = 43), and mutants (*n* = 53) groups. ***H***, Comparison of maximum tail bend angle of contralateral turns between the three groups (*n* = 140 trials, WT; *n* = 254 trials, HET; *n* = 281 trials, mutants). Kruskal–Wallis; Mann–Whitney test for between-groups comparisons with Bonferroni correction for multiple comparisons, *p < 0.025, **p < 0.005, ***p < 0.0005; ns, not significant.

Next, we asked whether *auts2a* mutants exhibited any deficits in escape behavior. We evoked the C-start escape behavior in partially restrained zebrafish larvae between 6 and 8 dpf, by directing a strong jet of water at the otic vesicle (OV; Fig. 3*A*). The restrained preparation ensures similar location of water jet delivery across larvae. The C-start escape response consists of a large angle contralateral tail bend initiated within 3–13 ms of stimulus delivery (Fig. 3*B*). We measured three parameters associated with the C-start escape response: the probability of initiating escapes (% trials where escapes were observed), the latency (time from stimulus arrival at the OV to movement onset) and the maximum tail bend angle, in wild type, heterozygotes, and *auts2a* mutant larvae. First, *auts2a* mutants showed a higher percentage of failures to initiate escape responses compared with heterozygotes and wild-type larvae (Fig. 3*C*). While wild-type and heterozygote larvae showed a contralateral tail bend response in nearly 98% of trials, *auts2a* mutants were able to generate escapes in only 76% of trials. Mutants also had increased probability of failures (no tail bend response) compared with heterozygote and wild-type larvae. Next, we asked whether the increased failure rates observed in mutants was because of few individuals that did not respond across any of the trials. Surprisingly, we observed that individual larvae displayed highly variable responses across trials (Movie 1). Figure 3*D* shows responses from five wild-type and five mutant larvae across six trials. While all wild-type larvae were able to initiate escape responses within 10–20 ms, four of five *auts2a* mutants showed failures in at least one trial. In addition, the latency to initiate escapes was longer and more variable in mutants compared with wild type (range: 3–552 ms; Fig. 3*D*,*E*). Escape response can be generated via multiple pathways. While fast escape responses to head directed stimuli result from activation of the M-cells and its segmental homologs (M-series; Liu and Fetcho, 1999; Kohashi and Oda, 2008), neural circuitry underlying long-latency escape responses is less well understood (but see Marquart et al., 2019). Therefore, we next compared latencies of the fast escape response (cutoff latency: ≤20 ms) between wild type, heterozygotes, and *auts2a* mutants. Fast escape responses in *auts2a* mutants occur at longer latencies compared with wild type (Fig. 3*F*). Further, the coefficient of variation (CV) of latencies for individual larvae across successive trials was significantly higher for the mutant group in comparison to wild-type larvae (Fig. 3*G*). Nevertheless, the kinematics of the escape response were not affected in mutants, as evidenced by the similar maximal tail bend angles observed (Fig. 3*H*). Thus, initiation of tightly regulated, high performance escape response is unreliable and slow in *auts2a* mutants.

### Escape response defects in *auts2a* mutants persist on changing the location of sensory stimulation

**Figure 4.**
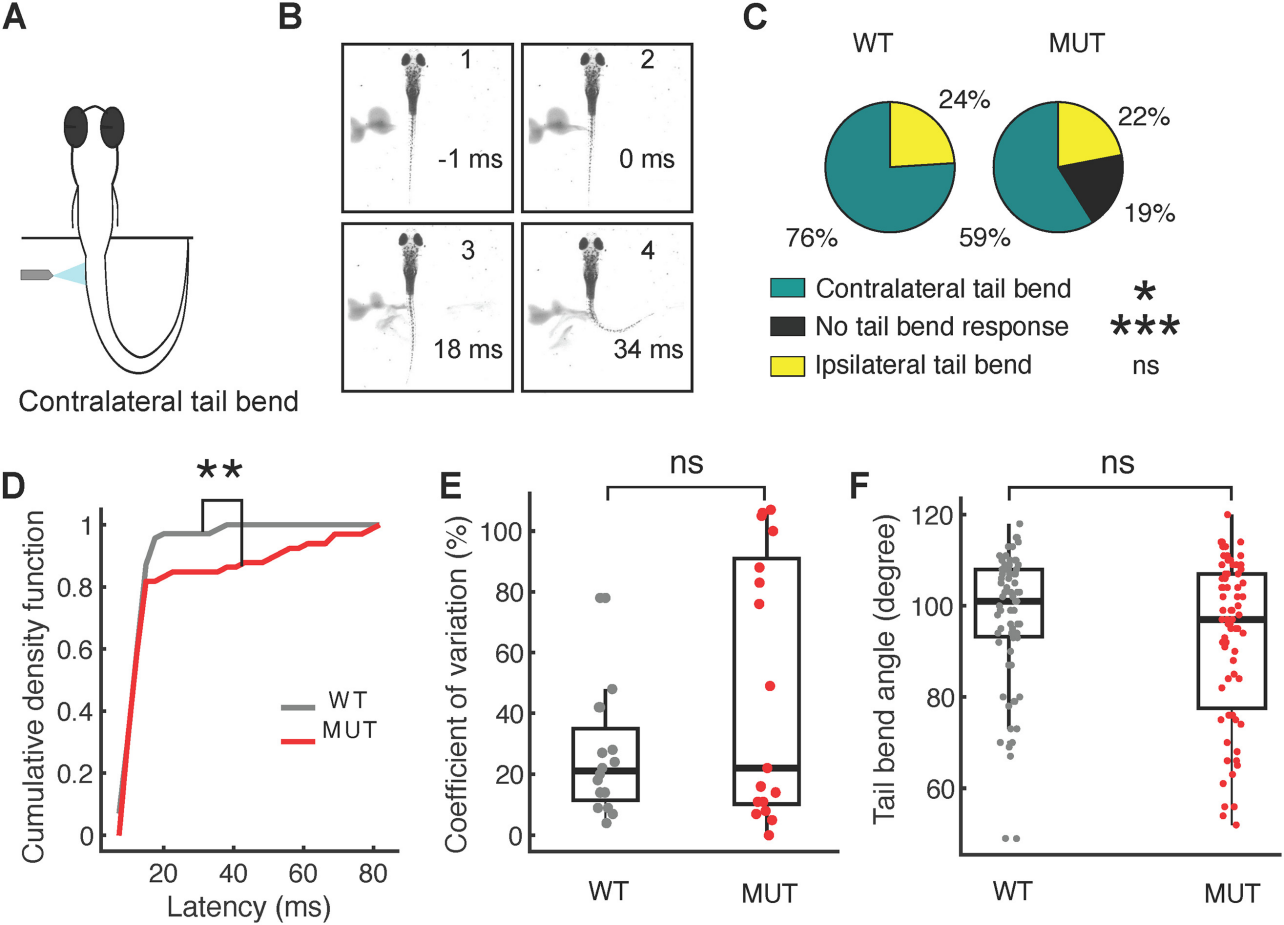
Escape response defects in *auts2a* mutants persist on changing the location of sensory stimulation. ***A***, Schematic of experimental set up. ***B***, Time lapse of escape response evoked by tail stimulation. (1) Prestimulus frame. (2) Water jet makes first contact with the tail. (3) First visible tail contraction (marked with asterisk). (4) Representative frame for maximum tail bend angle calculation. ***C***, Pie chart showing percentage of contralateral tail bends, ipsilateral tail bends, and failures to initiate an escape response between wild type (*n* = 92 trials) and mutants (*n* = 113 trials). ***D***, Comparison of escape response latencies on tail stimulation between wild type (*n* = 71 trials,16 larvae) and *auts2a* mutants (*n* = 67 trials, 19 larvae). ***E***, Comparison of CV of latencies across successive trials for each larva between wild-type (*n* = 17) and mutant (*n* = 18) groups. ***F***, Maximum tail bend angle of turns for WT (*n* = 72 trials) and mutants (*n* = 72 trials); *p < 0.05, **p < 0.005, ***p < 0.0001; ns, not significant.

Stimulation of the OV with water jet activates the M-cell and its homologs, while the same stimulus applied to the tail activates the M-cell alone (O’Malley et al., 1996; Liu and Fetcho, 1999). To investigate whether M-cell mediated escapes are compromised in *auts2a^ncb104^* larvae, we applied the water jet to the tail at the level of the cloaca (Fig. 4*A*,*B*). In wild-type larvae, tail stimulation with water pulse resulted in fast escape responses with characteristic short latency and contralateral tail bend in 76% trials (Fig. 4*B*,*C*). Similar to OV stimulation, *auts2a* mutants displayed a high percentage of failures in escape response but similar percentage of ipsilateral tail bend responses to wild type (Fig. 4*C*). Tail stimulation also resulted in significantly longer latencies in mutants than in wild-type larvae (Fig. 4*D*). However, no significant difference was observed in the CV of latencies across trials in an individual larva between wild-type and mutants (Fig. 4*E*). This result indirectly implies that the increased CV in latencies seen with the head stimulation of *auts2a* mutants (Fig. 3*G*) is contributed by deficits in M-cell homologs. No difference was observed in tail bend angles between wild-type and mutant groups (Fig. 4*F*).

### Mauthner neuron fails to fire reliably in *auts2a* mutants

**Figure 5.**
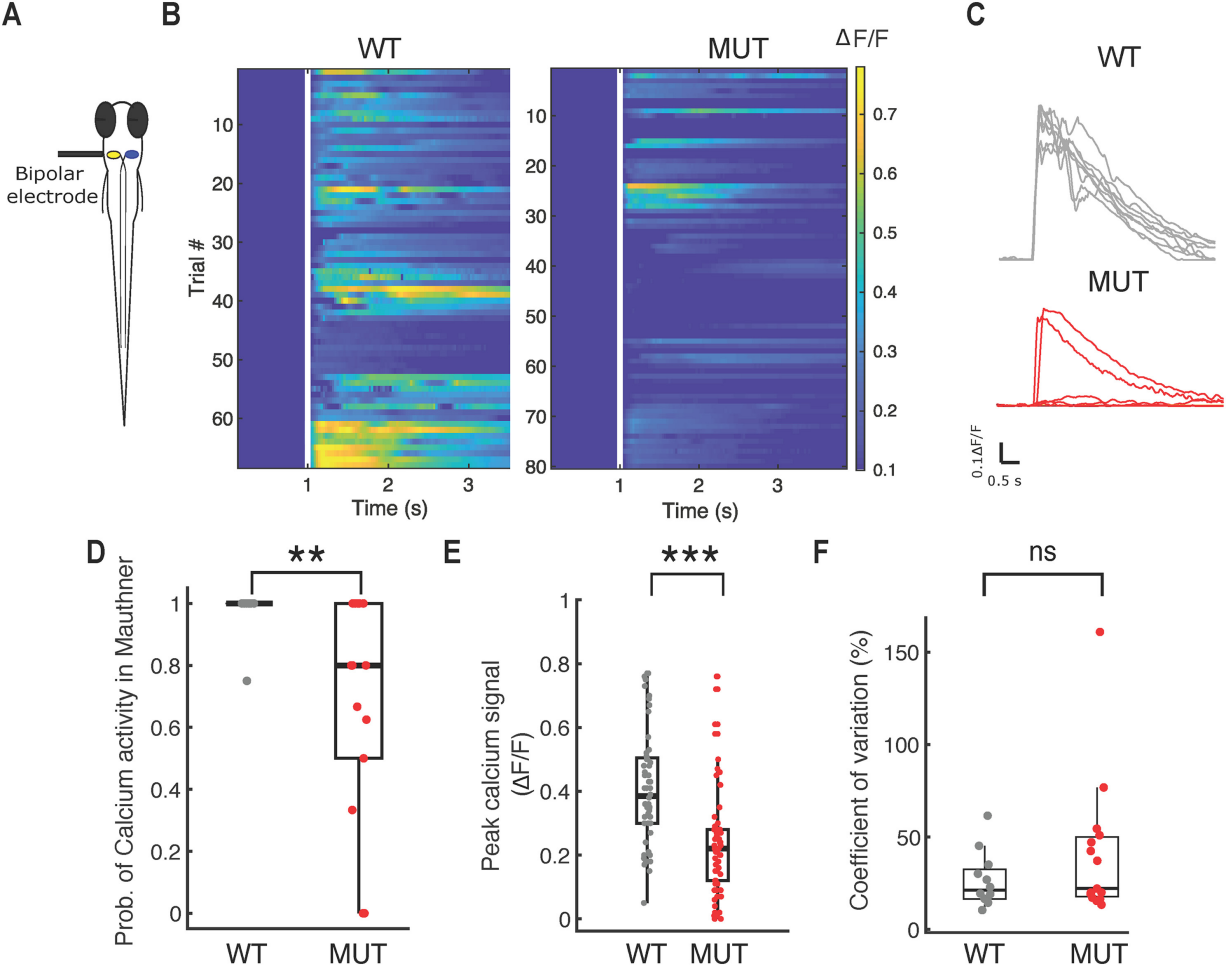
Mauthner neuron fails to fire reliably in *auts2a* mutants. ***A***, Schematic representation of experimental set up. Mauthner neuron was retrogradely labeled with OGB-1 dextran and calcium activity was monitored on electrical stimulation (40 μA, 1 ms) of OV. ***B***, left, Raster plot of all trials in WT (*n* = 68 trials; 10 larvae) showing consistent calcium activity across several trials on OV stimulation. Right, Calcium responses observed across all trials in the mutant group (80 trials; 14 larvae). White line represents the time of stimulus delivery. ***C***, top, ΔF/F profile of a Mauthner neuron in an example wild-type larva across eight trials in response to electrical stimulation of the OV. Bottom, ΔF/F profile of a Mauthner neuron in an example *auts2a* mutant larva showing subthreshold response as well large calcium transients across eight trials on electrical stimulation of OV. ***D***, Probability of calcium activity response across trials per larva (*n*_WT_ = 10 larvae, *n*_MUT_ = 14 larvae). ***E***, Peak ΔF/F in WT and mutants (*n*_WT_ = 68 trials, *n*_MUT_ = 80 trials). ***F***, Comparison of CV of peak ΔF/F between wild-type and mutant larvae. Mann–Whitney *U* test; **p < 0.01, ***p < 0.0001, ns: not significant.

To directly assess M-cell firing in *auts2a* mutants, we next monitored calcium activity in OGB-1 dextran labeled M-cells on electrical stimulation of OV (Fig. 5*A*). OV stimulation resulted in a large increase in fluorescence from rest (Fig. 5*B*,*C*; Movie 2) and this response was evoked reliably in wild-type larvae (Fig. 5*B–D*). Mutants displayed a greater proportion of failures in calcium response (Fig. 5*B–D*; Movie 3). Mutants also showed lower peak calcium signals compared with wild type (Fig. 5*C*,*E*). In addition, the CV of the peak calcium response was not statistically different between wild-type and mutant larvae (Fig. 5*F*). This implies that the increased variability in latency seen with OV stimulation (Fig. 3*G*) does not arise from variability in the response of the M-cell itself.

Movie 1. Escape responses in an auts2a mutant larva showing normal response (left), slow response (middle) and no response in three different trials. 10.1523/ENEURO.0493-20.2021.video.1

Movie 2. OGB-1 labeled Mauthner neuron in a wild-type larva showing robust calcium response after otic vesicle stimulation. 10.1523/ENEURO.0493-20.2021.video.2

Movie 3. OGB-1 labeled Mauthner neuron in an auts2a mutant larva showing failure to respond after otic vesicle stimulation. 10.1523/ENEURO.0493-20.2021.video.3

### Mauthner neurons in *auts2a* mutants have reduced excitability

**Figure 6.**
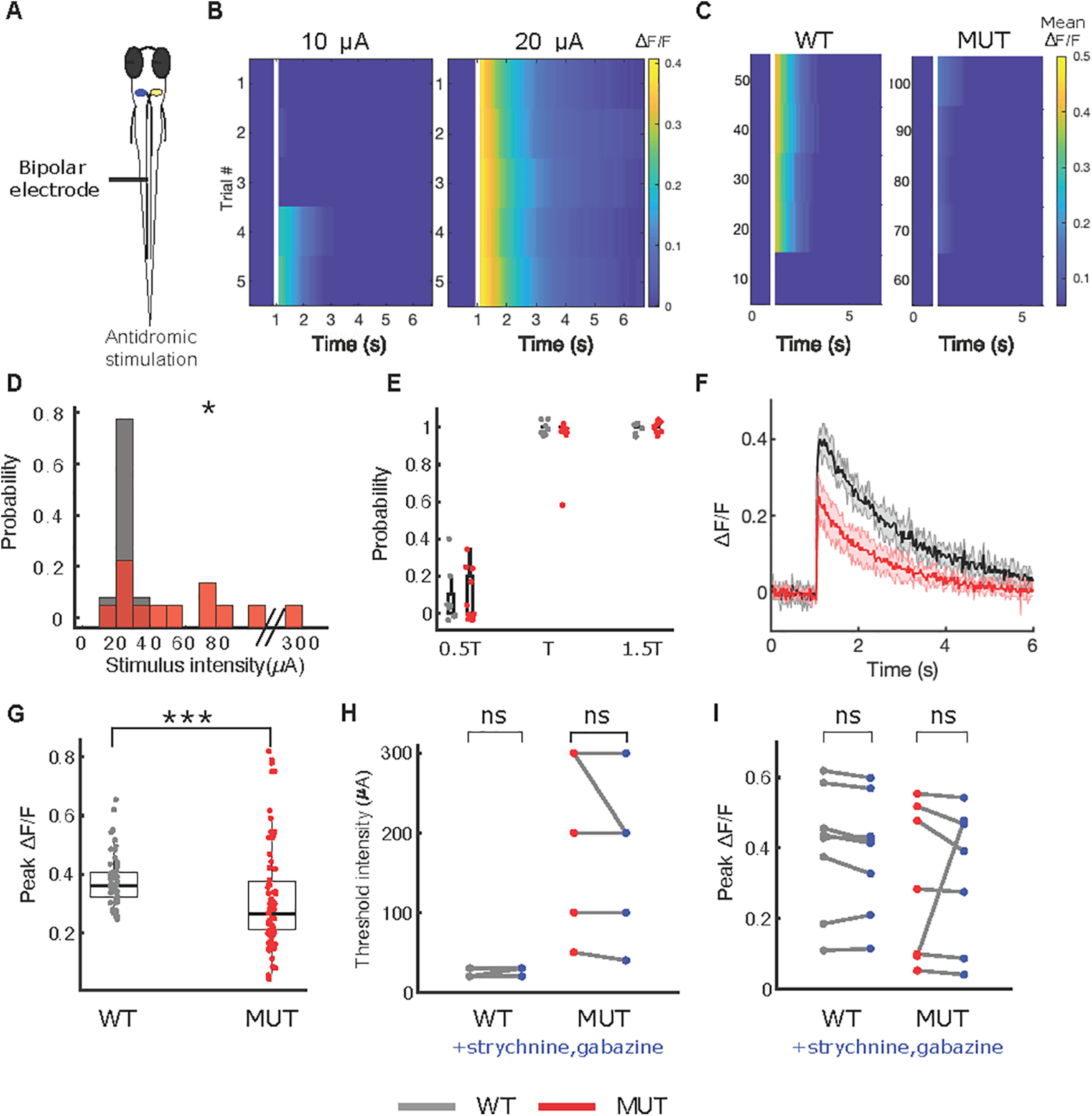
Mauthner neuron in *auts2a* mutants have reduced excitability. ***A***, Schematic of experimental set up. Calcium activity in the Mauthner neuron was observed on antidromic stimulation. Mauthner neuron was retrogradely labeled with OGB-1 dextran. ***B***, ΔF/F profile for an example wild-type (WT) larva on antidromic stimulation with 10-μA (left) and 20-μA (right) stimulus intensity. Mauthner neuron fired reliably at the threshold intensity of 20 μA. ***C***, Representative raster plot from a wild-type larva (left) and mutant larva (right). Each row represents average ΔF/F over five trials at the respective stimulus intensity. The threshold for calcium activity for wild-type larva is 20 μA, whereas for the mutant larva is 70 μA. ***D***, Normalized histogram of calcium activity threshold for wild type (*n* = 9 larvae) and *auts2a* mutants (*n* = 12 larvae). ***E***, Summary data of probability of calcium activity at 0.5×, 1×, 1.5× threshold stimulus intensity for wild-type and mutant group. ***F***, ΔF/F profiles for a representative wild-type larva (black) and a mutant larva (red) on antidromic stimulation. Shaded regions represent SEM from five trials. ***G***, Summary data of peak calcium signal in wild type (*n* = 45 trials, 9 larvae) and *auts2a* mutants (*n* = 60 trials, 12 larvae); *p < 0.05, ***p < 0.0005; ns: not significant; Mann–Whitney *U* test. ***H***, Calcium activity threshold for wild-type (*n* = 9) and mutant (*n* = 7) larvae before and after bath application of 50 μm strychnine and 100 μm gabazine. Mauthner neurons were labeled with calcium green dextran for this experiment. Wilcoxon signed-rank test. ***I***, Peak ΔF/F for wild-type (*n* = 9) and mutant (*n* = 7) larvae before and after bath application of 50 μm strychnine and 100 μm gabazine. Paired sample *t* test.

Failures in calcium activity response in the mutants after OV stimulation could result either from reduced excitability of M-cells and/or defects in sensory processing involving mechanosensory hair cells and the VIIIth cranial nerve. To ascertain the role of the M-cells, we stimulated its axon (antidromic stimulation) which resulted in calcium activity transients similar to OV stimulation (Fig. 6*A*).

Because of the large diameter of the M-cell axon, it has the lowest threshold for extracellular stimulation (Kimmel et al., 1982). At low stimulation intensity other cells are unlikely to be activated, making the stimulation M-cell specific. We defined the threshold intensity as the minimum stimulus intensity at which calcium signals were evoked in the M-cell in at least three out of five trials (Fig. 6*B*). Compared with wild type, threshold intensity was significantly higher in *auts2a* mutants (Fig. 6*C*,*D*). However, at intensities equal to or higher than threshold, M-cells responded reliably in both mutants and wild-type larvae (Fig. 6*E*) and the peak calcium signal was significantly reduced in mutants compared with wild-type larvae (Fig. 6*F*,*G*), similar to that seen after OV stimulation (Fig. 5*E*).

Alternatively, the increased threshold to fire in M-cells could result from increased inhibition on them. To rule out this possibility, we next performed antidromic stimulation before and after application of strychnine and gabazine to block glycine receptors and GABA-A receptors, respectively (Takahashi et al., 2002; Roy and Ali, 2014). Application of these antagonists did not alter the threshold intensity (Fig. 6*H*) or peak ΔF/F (Fig. 6*I*) in the wild-type or *auts2a* mutant larvae. These results show that the increased failures, latency, and variability in latencies all derive from increased threshold to fire M-cells.

## Discussion

**Figure 7.**
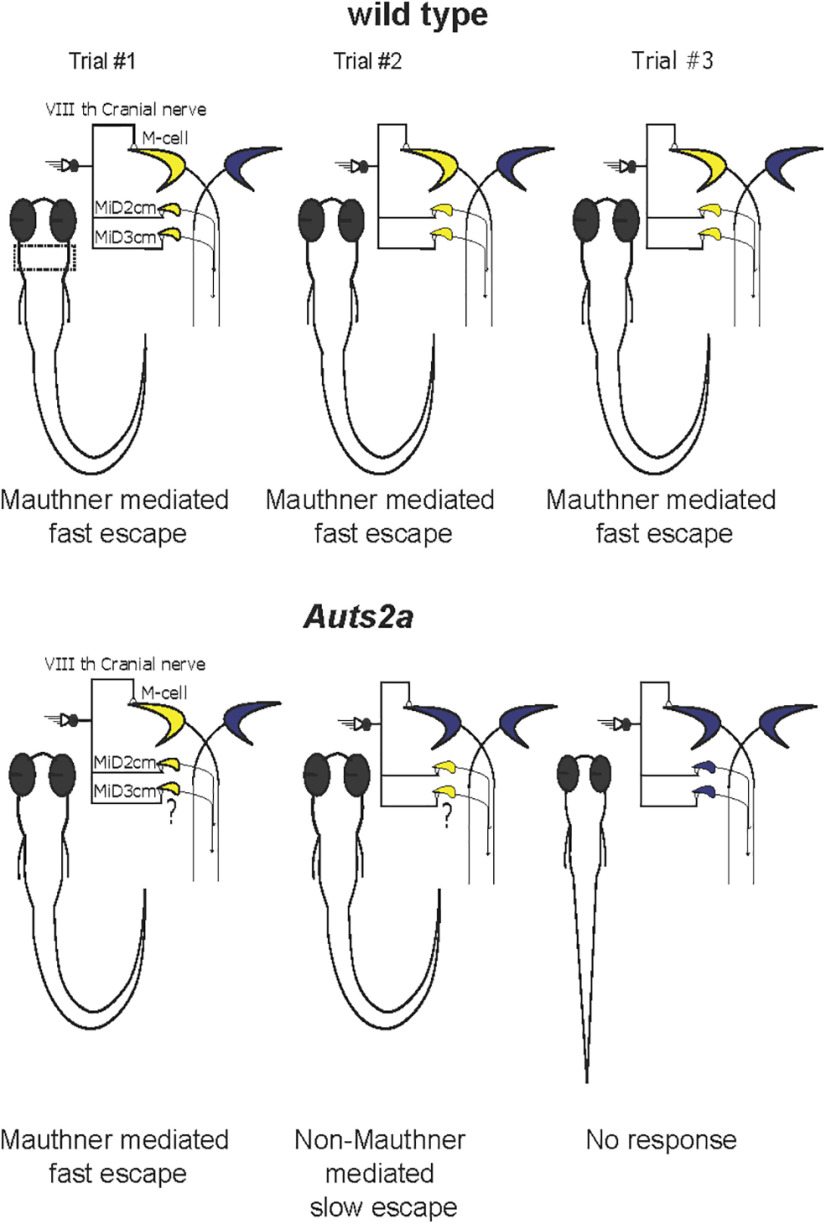
Summary of behavioral abnormalities in escape response in *auts2a* mutants. Top, In response to threatening stimuli, the ipsilateral Mauthner neuron and its homologs in the hindbrain (marked in a dashed box) fire reliably (yellow) resulting in short latency escape responses across consecutive trials (left to right) in wild-type larvae. Bottom, In *auts2a* mutants, Mauthner neurons fire unreliably. This means that on some trials, larvae exhibit normal short latency escapes when the Mauthner neuron is able to fire (left). On trials, where the Mauthner fails to fire, long latency escape responses may be initiated perhaps because of the activation of homologs (middle) and if neither the Mauthner, nor the homologs fire, then the larvae fail to respond (right). “?” denotes putative activity in Mauthner homologs during Mauthner-mediated and non-Mauthner-mediated escapes.

We show that *auts2a* mutant zebrafish exhibit interesting deficits in escape response: the response probability and latency are highly variable across trials in individual mutant larvae. As a consequence of *auts2a* mutation, reliable firing of M-cells is lost and therefore, escape responses become unreliable also. To explain the behavioral variability, we propose that in trials where the M-cell fires, normal short latency escape is triggered; in trials where neither the M-cell nor its homologs fire, fish fail to generate escape responses; and in trials where only the homologs fire, the latency to respond is much longer (Fig. 7). We confirmed the hypoexcitability of the mutant M-cells by directly stimulating the M-axon. Mounting an appropriate and fast escape response reliably is essential for survival and thus Auts2a serves an essential function for the animal. We also observed reduced peak ΔF/F calcium response in the mutants. As calcium response on antidromic stimulation of M-cells reflects calcium influx via voltage-activated calcium channels (Takahashi et al., 2002), the reduced peak calcium response could result from deficits in the number or activation of these channels in the mutants.

We also showed that somata and dendrites of *auts2a* mutant M-cells are smaller compared with wild-type. M-cells receive dense synaptic input on their dendrites and reduction in dendritic size may lead to decrease in synaptic drive. The smaller size of the cells may lead to reduced channel conductances, and/or reduced synaptic drive. In addition, global *auts2a* KO, as was done in this study, is indeed likely to result in widespread changes both within the escape network and outside. Nevertheless, given that we see longer, more variable escape latencies and a consistent increase in the threshold of M-cell firing in the *auts2a* mutants, we can conclude that these specific deficits in the short latency escape behavior are because of the hypoexcitability of the M-cell.

### The escape circuit as a model circuit for testing gene function

The escape system in zebrafish has been an advantageous tool for dissecting the genetic underpinnings of behavior, learning and decision-making (Gahtan and Baier, 2004; Wolman and Granato, 2012). Forward genetic screens identified mutants with specific deficits in generating escapes such as the *twitch twice* and *space cadet* mutants (Granato et al., 1996; Lorent et al., 2001; Burgess et al., 2009). More recent studies have reported mutants with deficits in sensitivity (Marsden et al., 2018), habituation (Wolman et al., 2015), prepulse inhibition (Burgess and Granato, 2007), or in deciding between Mauthner-mediated short latency escapes and non-Mauthner-mediated long latency escapes (Jain et al., 2018). The specific defects identified in these studies range from errors in axon guidance, extracellular calcium sensing and IGF signaling in M-cells and other members of the escape circuit. Our study makes an important addition to these studies by identifying Auts2a to be a direct genetic determinant of excitability in M-cells.

M-cells are specified soon after gastrulation and evoke escape responses to touch in larvae as young as early as 2 dpf (Kimmel et al., 1974; Kohashi et al., 2012). As the larvae mature, M-cells drive startle behaviors in response to auditory/vestibular stimulation as well. Concomitant with these changes, the firing behavior of M-cells changes from firing multiple action potentials on reaching threshold to firing only a single action potential after four dpf. This change in firing behavior of the M-cells drives maturation of the escape behavior from one involving multiple C-bends to that with only a single C-bend followed by routine swimming. The alteration in M-cell firing behavior is in part because of the expression of distinct types of potassium channels including those that are sensitive to dendrotoxin (Watanabe et al., 2014, 2017). These studies underline the critical importance of regulating the intrinsic properties of M-cells for generating appropriate C-start behaviors. Hyperexcitability will result in multiple C-starts while hypoexcitability in M-cells and its homologs, as seen in *auts2a* mutants, will lead to failures in escapes.

### Mechanism of action

ChIP-seq analysis revealed that in the mouse brain, AUTS2 binds preferentially to promoter and enhancer regions of genes involved in nervous system development (Gao et al., 2014; Oksenberg et al., 2014). In mouse forebrain alone, 784 AUTS2 binding sites were in promoter regions and 1146 sites were distal to promoter regions. Regardless of whether the sites were within promoter regions, these AUTS2 binding sites were found to be associated with genes implicated in ASDs. Further, binding of AUTS2 to these sites seems to activate their expression resulting in higher transcript levels. Importantly, among the targets of AUTS2 binding are genes associated with intracellular calcium homeostasis such as pumps and transporters, voltage-gated calcium channels and sodium channels, potassium channels as well as synaptic receptors (Oksenberg et al., 2014). If these targets are conserved in zebrafish as well, loss of Auts2a might interfere with expression levels of one or more of these targets leading to hypoexcitability of M-cells.

Manipulations that reduce global activity levels of neurons in culture lead to homeostatic resetting of intrinsic and synaptic properties (Turrigiano et al., 1998; Desai, 2003; Turrigiano and Nelson, 2004), a process that requires transcription (Ibata et al., 2008). Recently, it was shown that manipulations that induce homeostatic plasticity also trigger significant upregulation of AUTS2 expression (Schaukowitch et al., 2017). Thus, on the basis of our study and these earlier studies, we propose that Auts2 is important for setting and maintaining the excitability set-points of neurons.

### Alterations in neuronal excitability and ASDs

Since ASDs are frequently also comorbid with epilepsy, and because mutations that reduce inhibition or increase excitation frequently associate with them, ASDs were initially thought to result from hyperexcitation in the central nervous system (Rubenstein and Merzenich, 2003). However, it is becoming clear that balance between excitation and inhibition is key and when that balance is tilted toward more excitation or more inhibition, network function is impaired leading to ASD-like phenotypes (Nelson and Valakh, 2015; Lee et al., 2017; Sohal and Rubenstein, 2019). Activity imaging using expression of the immediate early gene *c-Fos* reveals hypoactivity in much of the forebrain in a mouse model of Rett syndrome (Kron et al., 2012). Reduced connectivity and reduced activation of forebrain structures at rest have also been reported in fMRI studies of human autistic subjects (Minshew and Keller, 2010). These studies underline the fact that since ASDs are associated with mutations in multiple genetic pathways, they should be thought of as diseases resulting from abnormal excitation to inhibition balance. Consistent with this view, a recent study demonstrates that in *Auts2* mutant mice, hippocampal pyramidal neurons receive increased excitatory synaptic inputs with no change in the amount of inhibition received, upsetting the excitation to inhibition balance (Hori et al., 2020). We have shown that loss of function of Auts2a in zebrafish leads to hypoexcitability in an identified neuron, the M-cell, which is responsible for driving fast escapes. Further, we showed that the hypoexcitability is not because of altered inhibition to the M-cells. Auts2a might affect M-cell firing by acting on its intrinsic properties and the targets remain to be identified in future studies.

## Synthesis

Reviewing Editor: Leonard Maler, University of Ottawa Decisions are customarily a result of the Reviewing Editor and the peer reviewers coming together and discussing their recommendations until a consensus is reached. When revisions are invited, a fact-based synthesis statement explaining their decision and outlining what is needed to prepare a revision will be listed below. The following reviewer(s) agreed to reveal their identity: Alberto Pereda, Tod Thiele.

Editor comments.

Dear authors,

I have included the comments of one reviewer.

This reviewer has made some good points.

I do not expect more experiments, but I think it would to note that there are some limitations to your interpretations of the data and then suggest future research directions that will extend your interesting discoveries.

I will simply do a Editorial check and will accept any reasonable revisions that discuss the points raised by this reviewer

Thank you and hope to get you published soon

Leonard Maler

Reviewer comments

I appreciate the efforts made by the authors to address the concerns raised in the previous review cycle. However, I remain skeptical about some of the authors’ claims and conclusions. Here are my thoughts on the paper:

• I am totally convinced that the auts2 mutation leads to deficits in the M-cell network, as evidenced by the smaller M-cell.

• Despite that the smaller size of the M-cell is the most evident consequence of the mutation, anatomical differences with WT are not nicely documented and illustrated with convincing images regarding the size of their soma and dendrites. Reconstructions of the cells using Imaris would have been very informative.

• Also, smaller M-cells is auts2 could explain the weaker Ca++ response in the absence of changes in excitability. Shouldn’t Ca++ responses be normalized to cell size for better comparison between mutants and WT?

• If the M-cells show morphological changes in auts2 mutants, it is also likely that its inputs are developmentally altered, potentially leading to altered excitatory/inhibitory balance. This possibility is supported by the reported smaller size of the dendrites receiving most of these inputs.

• Thus, rather than circumscribed to the M-cell excitability, changes in auts2 mutants might be widespread. Variability of responses and threshold can be explained by widespread deficits involving sensory excitatory inputs, inhibitory inputs and M-cell excitability.

• Testing as suggested the overall excitatory drive to the M-cell is not possible, as the authors claim that they cannot monitor with their system subthreshold Ca++ responses in the soma and lateral dendrite (as done in Marsden and Granato, Cell Rep, 2015) in response to OV stimulation, leaving this possibility unanswered.

• I raised concerns in my previous review about a possible modification of the strength of inhibition as a potential mechanism for changes in M-cell excitability. The authors performed experiments in the presence of strychnine and gabazine that, they claim, allow them to rule out this possibility. However, the experiments lack of a positive control for the effect of the drugs. Moreover, along these lines, blocking inhibition should have led to an increase in Ca++ responses, as shown in Marsden and Granato, Cell Rep. 2015 (Fig. 4B) and Takahashi et al. J Neurosc. 2002 (Fig. 7). Thus, potential changes in inhibition also remain untested.

• As also suggested previously, a more direct way to test the strength of inhibition is the method use by Takahashi et al. J Neurosc. 2002 (Figs. 4-7) using double stimulation at different intervals. This was a much better approach.

In summary, I remain unconvinced of the authors’ claim that deficits can be only, or mainly, explained by changes in the excitability of the M-cell.

## Author Response

Response to Reviewer’s comments:

---------------------------------------------

Reviewer comments

• I am totally convinced that the auts2 mutation leads to deficits in the M-cell network, as evidenced by the smaller M-cell.

Thank you.

• Despite that the smaller size of the M-cell is the most evident consequence of the mutation, anatomical differences with WT are not nicely documented and illustrated with convincing images regarding the size of their soma and dendrites. Reconstructions of the cells using Imaris would have been very informative.

We performed the measurements of soma and dendrite size using Fiji. We are not sure what additional information could have been obtained with Imaris.

• Also, smaller M-cells is auts2 could explain the weaker Ca++ response in the absence of changes in excitability. Shouldn’t Ca++ responses be normalized to cell size for better comparison between mutants and WT?

The calcium responses were measured after backfilling with calcium sensitive dyes OGB-1 or CGD while the cell morphometry was done after back-filling with TMR-dextran as this dye fixes well and is brighter. Since the datasets were obtained from different M-cells, we cannot normalize the Ca2+ responses by cell volume. However, following this reviewer’s suggestion, we normalized dF/F with respect to cross-sectional area of the M-cell being imaged. The results were not altered after normalization and the peak dF/F in auts2 mutants continued to be significantly lower than that seen in wild type (p<0.0001).

We decided to leave the figure as is in the manuscript, as dF/F values are usually reported as percentage values or a number between 0-1. The small y-axis values seen above, due to the normalization, might needlessly confuse readers.

• If the M-cells show morphological changes in auts2 mutants, it is also likely that its inputs are developmentally altered, potentially leading to altered excitatory/inhibitory balance. This possibility is supported by the reported smaller size of the dendrites receiving most of these inputs.

• Thus, rather than circumscribed to the M-cell excitability, changes in auts2 mutants might be widespread. Variability of responses and threshold can be explained by widespread deficits involving sensory excitatory inputs, inhibitory inputs and M-cell excitability.

• Testing as suggested the overall excitatory drive to the M-cell is not possible, as the authors claim that they cannot monitor with their system subthreshold Ca++ responses in the soma and lateral dendrite (as done in Marsden and Granato, Cell Rep, 2015) in response to OV stimulation, leaving this possibility unanswered.

In response to the three points above, we include the following sentences in the discussion to accommodate these limitations: (lines 422-431)

” We also showed that somata and dendrites of auts2 mutant M-cells are smaller compared to wild type. M-cells receive dense synaptic input on their dendrites and reduction in dendritic size may lead to decrease in synaptic drive. The smaller size of the cells may lead to reduced channel conductances, and/or reduced synaptic drive. Global auts2 knock-out, as was done in this study, is indeed likely to result in widespread changes both within the escape network and outside. Nevertheless, given that we see an increase in the threshold of M-cell firing in the auts2 mutants, and consistent with it, longer, more variable escape latencies, we can conclude that these specific deficits in the short latency escape behavior are due to the hypoexcitability of the M-cell. “

• I raised concerns in my previous review about a possible modification of the strength of inhibition as a potential mechanism for changes in M-cell excitability. The authors performed experiments in the presence of strychnine and gabazine that, they claim, allow them to rule out this possibility. However, the experiments lack of a positive control for the effect of the drugs.

These drugs are regularly made fresh and used in our lab and recordings from other neural types conducted during the same time using the same aliquots show a complete abolition of IPSCs.

Moreover, along these lines, blocking inhibition should have led to an increase in Ca++ responses, as shown in Marsden and Granato, Cell Rep. 2015 (Fig. 4B) and Takahashi et al. J Neurosc. 2002 (Fig. 7). Thus, potential changes in inhibition also remain untested.

Actually, in Takahashi et al., 2002, the increase in peak dF/F after strychnine application was only reported for the second stimulus in a double stimulation experiment. No comparison for the peak dF/F for the first stimulus was given. From the figures, the peak dF/F for the first stimulus look comparable. Since we only use single stimulus pulses, the comparison should not be made with responses to double stimuli, which recruit inhibition.

As for Marsden and Granato, 2015, acoustic stimuli were delivered and not antidromic stimulation like we have done. While AD stimulation directly fires the M-cell, acoustic stimulation recruits sensory neurons, with longer synaptic delays and therefore likelihood for feed-forward and recurrent inhibition to have influences on peak dF/F. Thus the data from both papers are not comparable one-to-one with our data. We disagree with this reviewer’s view that the changes in inhibition remain untested.

• As also suggested previously, a more direct way to test the strength of inhibition is the method use by Takahashi et al. J Neurosc. 2002 (Figs. 4-7) using double stimulation at different intervals. This was a much better approach.

Takahashi et al., 2002 used double stimuli to show the effects of recurrent, feed-forward and contralateral inhibition on M-cell activity. We wanted to test if the increased threshold observed in auts2 mutants after single antidromic stimulation could be attributed to increased inhibition. Using double stimulation in this case will not answer this question.
