# Supplementary figures and images for: High Behavioral Variability Mediated by Altered Neuronal Excitability in auts2 Mutant Zebrafish
Source: eNeuro. 2021 Oct 8;8(5):ENEURO.0493-20.2021. doi: 10.1523/ENEURO.0493-20.2021 (PMC8503961; doi:10.1523/ENEURO.0493-20.2021)

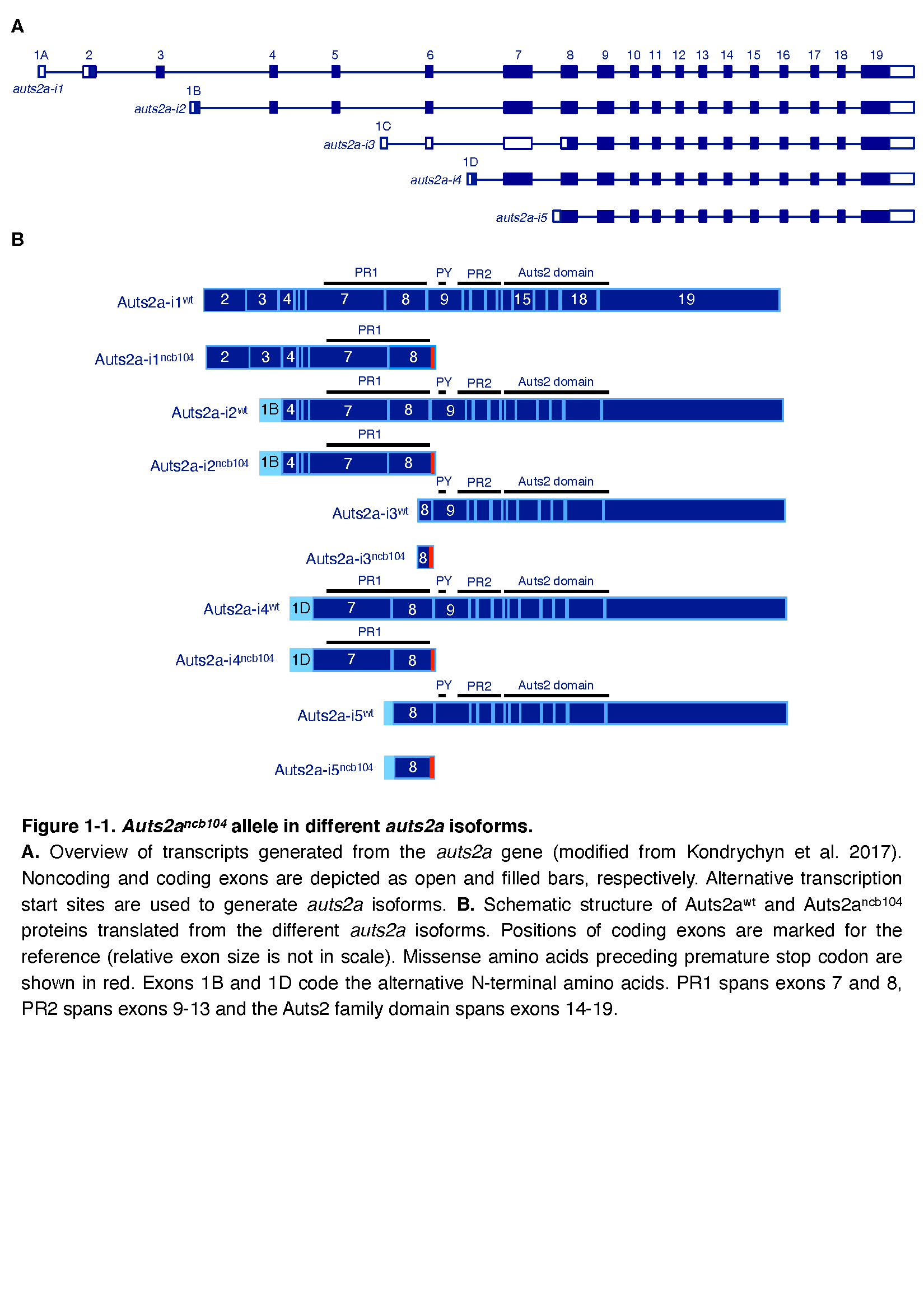

Supplement: Extended Data Figure 1-1 — Auts2ancb104 allele in different auts2a isoforms. A, Overview of transcripts generated from the auts2a gene (modified from Kondrychyn et al., 2017). Noncoding and coding exons are depicted as open and filled bars, respectively. Alternative transcription start sites are used to generate auts2a isoforms. B, Schematic structure of Auts2awt and Auts2ancb104 proteins translated from the different auts2a isoforms. Positions of coding exons are marked for the reference (relative exon size is not in scale). Missense amino acids preceding premature stop codon are shown in red. Exons 1B and 1D code the alternative N-terminal amino acids. PR1 spans exons 7 and 8, PR2 spans exons 9–13, and the Auts2 family domain spans exons 14–19. Download Figure 1-1, TIF file. [file enu-eN-NWR-0493-20-s04.tif]
